# An Efficient and QoS Supported Multichannel MAC Protocol for Vehicular Ad Hoc Networks

**DOI:** 10.3390/s17102293

**Published:** 2017-10-09

**Authors:** Caixia Song, Guozhen Tan, Chao Yu

**Affiliations:** 1 College of Computer Science and Technology, Dalian University of Technology, Dalian 116024, China; gztan@dlut.edu.cn (G.T.); cy496@dlut.edu.cn (C.Y.); 2 College of Science and Information, Qingdao Agricultural University, Qingdao 266109, China

**Keywords:** Vehicular Ad Hoc Networks (VANETs), Multichannel Medium Access Control (MAC), transport efficiency and infotainment applications, service channel (SCH) reservation, Quality-of-Service (QoS) support

## Abstract

Vehicular Ad Hoc Networks (VANETs) employ multichannel to provide a variety of safety and non-safety (transport efficiency and infotainment) applications, based on the IEEE 802.11p and IEEE 1609.4 protocols. Different types of applications require different levels Quality-of-Service (QoS) support. Recently, transport efficiency and infotainment applications (e.g., electronic map download and Internet access) have received more and more attention, and this kind of applications is expected to become a big market driver in a near future. In this paper, we propose an Efficient and QoS supported Multichannel Medium Access Control (EQM-MAC) protocol for VANETs in a highway environment. The EQM-MAC protocol utilizes the service channel resources for non-safety message transmissions during the whole synchronization interval, and it dynamically adjusts minimum contention window size for different non-safety services according to the traffic conditions. Theoretical model analysis and extensive simulation results show that the EQM-MAC protocol can support QoS services, while ensuring the high saturation throughput and low transmission delay for non-safety applications.

## 1. Introduction

Vehicular Ad Hoc Networks (VANETs) are part of the Intelligent Transportation System (ITS), which aim to provide safety-critical and commercial services on the road. VENETs can perform Vehicle To Vehicle (V2V) and Vehicle To Infrastructure (V2I) communications by On Board Units (OBUs) and Road Side Units (RSUs). Through V2V and V2I communications, vehicles can exchange information to support safety-related applications (e.g., emergency brake, collision avoidance, and automatic notification of crash on roads), transport efficiency applications (e.g., intersection management, navigation and lane merging assistance, etc.) and infotainment applications (e.g., voice over IP, video, web browsing and mobile multiplayer gaming, etc.) [1,2,3]. The transport efficiency applications and infotainment applications are regarded as non-safety applications.

Different applications have different Quality-of-Service (QoS) requirements. The traffic of safety applications has stringent requirements on highly reliable and real-time transmissions, while the non-safety applications require efficient and high throughput. Recent works [4,5,6,7] manifest that non-safety applications can have different communication requirements. On one hand, in terms of throughput, video and mobile multiplayer gaming need higher throughput than that of lane merging assistance [4,5]. On the other hand, non-safety applications also have different delay requirements, from no special real-time requirements of traveller information support applications (e.g., points of interest advertisements, map download) to guaranteed near-real time needs of some interactive entertainment applications (e.g., mobile multiplayer gaming and voice over IP) [6,7,8]. Providing QoS support for non-safety applications has the great potential to increase the chance of success for VANETs and to accelerate their market penetration [4]. Due to the wide variety of ITS applications, Medium Access Control (MAC) protocols need to be able to support a wide range of QoS requirements.

Wireless Access in Vehicular Environments (WAVE) is a protocol for the VANETs. WAVE includes IEEE 802.11p [9] and IEEE 1609.4 [10] protocols. In IEEE 802.11p protocol, Carrier Sense Multiple Access/Collision Avoidance (CSMA/CA) MAC mechanism is employed which is based on the prioritized Enhanced Distributed Channel Access (EDCA)—IEEE 802.11e protocol [11], to provide QoS for different ITS applications. WAVE defines seven channels: one Control Channel (CCH) and six Service Channels (SCHs). The multichannel MAC architecture and operation are specified in the IEEE 1609.4 protocol. In IEEE 1609.4 protocol, the channel time is divided into multiple Synchronization Intervals (SIs) with a fixed length of 100 ms for each SI. An SI consists of a 50 ms CCH Interval (CCHI) and a 50 ms SCH Interval (SCHI). During the CCHI, all vehicles need tune to CCH for the transmissions of safety-related messages or control messages such as WAVE Service Advertisement (WSA) messages, any other kind of communications which is running on the SCHs must be frozen, and vice versa.

However, the 802.11p contention-based access and the fixed channel switching defined in the IEEE 1609.4 make high throughput and time-bounded data delivery very difficult to be ensured in such a system [12,13]. On one hand, EDCA mechanism originally provided by IEEE 802.11e [11] that differentiates traffic types is based on different static MAC parameter values which can not adapt to the characteristics of ever-changing vehicle density. On the other hand, for example, in a high density VANETs environment, the nodes may not have enough time to perform effective negotiations and make SCH reservations on a highly congested CCH, and thus the utilization of SCHs is affected. On the contrary, under a VANETs environment with light vehicle density but requiring heavy network service (e.g., near points of interests), CCHI may be idle for a long time, while the 50 ms SCHI is not enough to transmit bulk and near-real time flows, like QoS-sensitive video/audio traffic, typical of many infotainment applications [4], which could be strongly penalized nonetheless their high channel access priority. Therefore, the all traffic experience the low throughput and additional unavoidable delay due to the WAVE channel switching procedure. The SCH resource is also wasted. In addition, when the number of nodes is sufficiently large, the maximum achievable aggregate throughput of the network depends on the number of channels, but not the number of nodes [14]. So, the number of SCHs and the duration on the SCHs are critical factors affecting the system throughput.

In [15], the length of the CCH interval is dynamically adjusted according to the number of vehicles, and the interval and channel are thus used efficiently. However, the packet collisions of the safety messages may not be mitigated in dense traffic. In [16], Time Division Multiple Access (TDMA)-based safety message transmission was proposed. With the help of RSU, nodes can use less time to transmit safety messages without contention and perform SCH reservation on CCH during CCHI, and thus leave more time for non-safety message transmission. In fact, during the CCHI, all SCHs are idle, therefore, the SCH resources are underutilized. By assigning disjoint time slots to the vehicles with different positions and directions, the collision problem of safety message transmission is mitigated in a moving-vehicle environment [17]. The nodes transmit non-safety messages on SCHs while the nodes transmitting safety messages based on TDMA mechanism on CCH. Therefore, the protocol in [17] can ensure the reliable transmissions for safety messages and high throughput for non-safety messages. Since additional information for slot occupancy is required, more time for broadcasting safety messages is needed, and consequently, the time left for SCH reservation is less. Therefore, the improvement of throughput of non-safety messages may be still limited. By dynamically adjusting the Contention Window (CW) size, the work in [5] can provide different QoS levels for kinds of non-safety applications with different priorities on the SCHs. However, the SCHs are idle when the nodes transmit messages on the CCH, the SCHs are still underutilized.

In this paper, we propose an Efficient and QoS supported Multichannel MAC (EQM-MAC) protocol, which is specifically targeted to provide high throughput, low delay and differentiated treatment to non-safety applications. In the EQM-MAC protocol, the non-safety messages can be transmitted over the whole SI. Therefore, the system saturation throughput and the utilization of SCHs can be greatly enhanced. The EQM-MAC protocol dynamically adjusts the CWs of WSA packets to achieve the predefined throughput ratio between non-safety packets with different priorities according to the traffic density. Therefore, the QoS delivery of non-safety packets is ensured.

The main feature of the EQM-MAC protocol and thus the contributions of this paper can be summarized as follows:

(1) The EQM-MAC protocol uses less time to deliver safety messages and allocates more time to make time slot reservations and channel coordination for SCHs. Therefore, nodes have more opportunities to perform SCH reservation to deliver different service classes packets, and the number of successful reservations can be greatly increased.
(2) The non-safety messages can be simultaneously transmitted on SCHs during the whole SI. Therefore, the saturation throughput and the utilization of SCHs can be further increased.
(3) EQM-MAC protocol can offer sufficient QoS in terms of throughput and delay for non-safety messages through adjusting the minimum CW according to the vehicle density.

The rest of this paper is organized as follows: Section 2 reviews related work. Section 3 describes the proposed EQM-MAC protocol in detail. Section 4 first presents a Markov chain model to analyze the transmission probabilities for WSA messages with different priorities, so as to drive the mean reservation time for SCHs, and then conducts performance analysis of throughput and delay. Simulation evaluation is given in Section 5. Finally, Section 6 concludes this paper.

## 2. Related Works

There are many works aim to provide reliable and real-time delivery of safety messages and efficient throughput of non-safety messages under multichannel vehicular environments. Some alternating (also called split-phase) channel access schemes have been proposed [15,16,18,19]. The authors in [15] proposed a Variable CCH Interval (VCI) multichannel MAC protocol to dynamically adjust the length of CCHI according to the number of vehicles on the road. The VCI protocol can maintain the prioritized transmission of critical safety messages and help enhancing saturated throughput of SCHs compared to IEEE 1609.4 protocol. An Adaptive multi-Priority Distributed Multichannel (APDM) MAC protocol is presented in [18]. The APDM protocol uses two Markov chain analytical models to achieve the optimal transmission probabilities for safety message with high priority and WSA with low priority. An Application Oriented Cross-layer Multi-channel (AOCM) MAC protocol was proposed for VANETs in [19]. Based on real-time statistics and prediction for different types of messages coming from upper application layer, the AOCM MAC protocol, with low computational complexity, can derive the optimal CCHI to ensure timely and reliable transmission of safety-related messages and enhance the throughput of SCHs. The work [16] proposed a Coordinated multichannel MAC (C-MAC) protocol. With the help of RSU, C-MAC can decrease the overhead of the reservation slots for safety messages, lower the collision probability of safety messages and improve the throughput of service channels. All the above protocols employ alternating channel access schemes, however, the SCHs are idle during the transmission of safety and control messages, resulting in an inefficient utilization of bandwidth of SCHs.

In fact, most multichannel schemes suffer from underutilization of SCHs [20,21]. This condition is often associated with the fact that nodes have very little time to make SCH reservations or when transmitting the safety-related messages, all the nodes switch to the CCH and the SCH is idle or both of the above cases. The authors in [20] proposed Asynchronous Multi-Channel Medium Access Control with a Distributed time-division multiple-access mechanism (AMCMAC-D). The AMCMAC-D scheme supports simultaneous disseminations on different SCHs while allowing rendezvous and broadcasting of safety messages on the CCH. The proposed scheme allows messages with different priorities having different numbers of time slots. Thus the utilization of the CCH and SCHs can be improved. However, in the AMCMAC-D scheme, the safety messages employ CSMA/CA mechanism to disseminate which is still a contention-based mechanism, and more time is required to transmit the safety messages especially under heavy vehicle density. Therefore, the nodes have less opportunities to make SCH reservations, and AMCMAC-D scheme cannot take full advantage of channel resources. A Coordinated Reliable and Efficient multichannel MAC (CRE-MAC) protocol for VANETs is proposed in [22], to meet requirements of delay-sensitive safety applications and throughput-sensitive non-safety applications. The CRE-MAC protocol employs contention-free transmission for safety messages and allows transmission of non-safety messages during a whole SI. Therefore, the reliable and timely transmission of safety messages, and the network throughput of non-safety messages are greatly improved.

All above the protocols (schemes) take the non-safety messages transmitted over the SCHs with the same priority and thus these protocols (schemes) cannot provide QoS supported for the non-safety applications with different priorities.

There are works focusing on studying the QoS mechanisms to provide guaranteed non-safety services to on-board passengers from the RSU [23,24]. The work [23] proposed a comprehensive analysis model taking into account both the QoS features and the vehicle mobility to seek solutions to optimally adjust the parameters towards the controllable QoS provision to vehicle. The work [24] provided a scheduling algorithm incorporating with EDCA to provision controlled QoS to vehicles. The scheduling algorithm controls TXOP of vehicles iteratively to maximize the integrated throughput according to the current queue length and packet error rate. In contrast to [23,24], our proposed EQM-MAC protocol works on the multichannel environments and focus on providing different proportions throughput for non-safety packets with different priorities. There are studies [5,25], which work under multichannel environments to provide different QoS levels for non-safety applications with different priorities on the SCHs. Ref. [5] adaptively tunes the CW for different services at each nodes and dynamically adjusts the CCHI to obtain the CW and optimal CCHI based on the traffic conditions. [25] presents a dedicated multichannel MAC scheme with QoS-provision channel allocation mechanism which is based on the EDCA channel throughput analysis to enhance the QoS performance of non-safety services. However, in above both schemes, the SCHs are idle when the safety packets are delivering on the CCH, and thus, the throughput of SCHs is reduced and the SCHs are still under utilization.

## 3. Efficient and QoS Supported Multichannel MAC Protocol

In this paper, we propose an Efficient and QoS supported Multichannel MAC (EQM-MAC) protocol, which is specifically targeted to provide high throughput, low delay and QoS differentiation for non-safety applications. A summary of the important notations used in this paper is given in Table 1.

**Figure 1:**
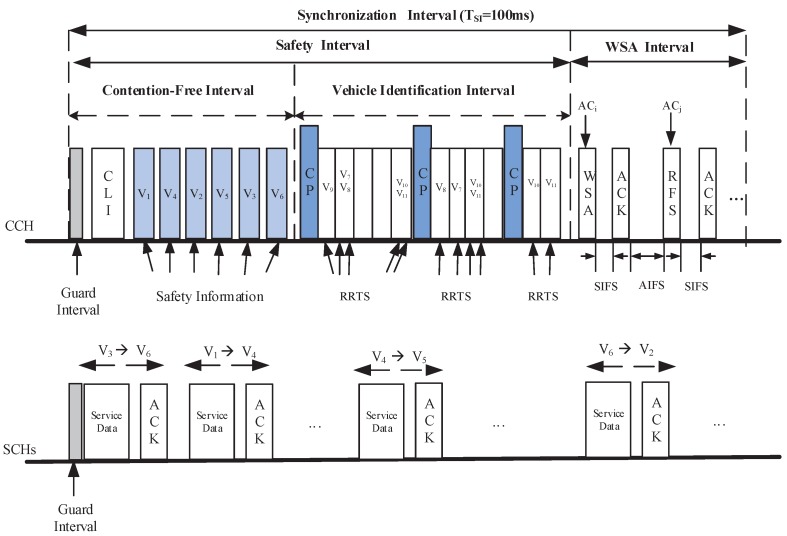
The framework of EQM-MAC protocol.

In our proposed EQM-MAC protocol, each vehicle is equipped with two transceivers (Note that the cost of two transceivers is practically trivial, as compared to the cost of the vehicle itself), which are denoted by Transceiver I and Transceiver II, respectively, which can operate simultaneously on different channels. Transceiver I is always tuned on the CCH while Transceiver II can be tuned to any SCH. The Coordinated Universal Time (UTC) [10] mechanism is used for time synchronization among all vehicles by Global Position System (GPS). Time is divided into multiple synchronization intervals with 100 ms for each SI, as shown in Figure 1.

On CCH, an synchronization interval includes two intervals: *Safety interval* and *WSA interval*. The *Safety interval* is used to service the safety-related messages, while in *WSA interval*, nodes conduct statistics and measurement for channel coordination and channel assignment. The *Safety interval* is further divided into two intervals [22]: Contention-Free Interval (CFI) and Vehicle Identification Interval (VII). A new synchronization interval begins from the CFI, during which RSU broadcasts a Coordination and Length Information (CLI) packet, and the vehicle nodes subsequently transmit the safety (e.g., beacon/emergency ) messages. The CLI packet contains the scheduling information for safety slots during the CFI, the minimum CW size for four Access Categories (ACs) (also called service classes in this paper), $T_{CFI}$, $T_{VII}$ and $T_{WI}$, where $T_{CFI}$, $T_{VII}$ and $T_{WI}$ denote the length of CFI, VII and *WSA interval*, respectively. For the sake of reliable delivery, each CLI packet will be broadcast twice. Each node knows its transmission order during CFI by receiving the CLI packet. During the VII, each new arriving vehicle derives a safety slot using DFSA [16,22,26]. The identity of each node is distinguished by a MAC address as well as a short IDentifier (ID), which serves to reduce the overhead for transmitting a CLI packet. The ID is chosen by each node at random, included in the header of each packet transmitted on CCH, and changed if the node detects that its ID is already in use by another node [17,27]. During the *WSA interval*, nodes make negotiations and reserve the SCHs for non-safety message transmissions for the next synchronization interval. After the negotiation is successful, in the next synchronization interval, the service provider and the service user switch to the SCHs that they have agreed over to complete the non-safety message transmission.

### 3.1. SCH Selection and Access Reservation Scheme

As shown in Table 2, each vehicle maintains a SCH Usage List (SUL). The SUL stores the available slots on each SCH of the next synchronization interval. According to the SUL, nodes make negotiations and reserve the SCHs by two-way WSA/Request For Service (RFS) handshake for the next synchronization interval. Each service provider sends a WSA packet, containing the service information, the AC and the selected [SCH, slot] to be used, as well as other information [10]. When a node has non-safety messages to deliver, it will select a slot of the corresponding SCH according to its SUL, and then, during the *WSA interval*, it employs CSMA/CA mechanism to contend the CCH for delivering the WSA message including its selected [SCH, slot]. When the receiver receives the intended WSA message, the receiver sends an Acknowledgement (ACK) message to the sender, if the [SCH, slot] is available for the receiver, or with a Non-acknowledgement (NACK) packet otherwise. Each service user can also initiatively broadcast an RFS packet associated with the selected [SCH, slot] to make an agreement with a service provider. To ensure load balancing of SCHs, the node selects the SCH that accommodates the most available slots in its SUL. If more than one SCH is available, the sender preferentially selects the same SCH used in the previous non-safety message transmission. On the other hand, to enhance the utilization of SCHs, EQM-MAC protocol allows the nodes to reserve multiple times, which means that the non-safety packets can be transmitted multiple times on SCHs during an synchronization interval. Since the transceiver I always senses and monitors the CCH, the multichannel hidden terminal problem [22] and miss-receiver problem [28] can be avoid.

### 3.2. Analysis of Differentiated Minimum Contention Window

In our study, we give a tractable yet reasonable model to characterize the performance of the proposed EQM-MAC protocol. In our model, we give the assumptions as follows:

**Assumption** **1** (Poisson distribution of vehicles on load). *Statistic analysis of the empirical data in [29] proves that an exponential distribution is a good fit for highway vehicle traffic according to inter-vehicle distance. Assuming that the vehicle nodes are placed on the line according to a Poisson point process with network density β (in nodes per meter), the probability of j vehicles existing within length l, P(j,l), can be given by*

(1) $$P\left(j,l\right)=\frac{{\left(\beta l\right)}^{j}e^{-\beta l}}{j!}$$

*Then the average number of vehicles, N, within the RSU coverage R is βR.*

**Assumption** **2** (Uniform distribution of vehicle speed). In each lane of each direction, the speed of vehicles has uniform distribution between $V_{min}$ and $V_{max}$ with mean $V_{avg}=\frac{V_{\min}+V_{\max}}{2}$ and variance $\phi^{2}=\frac{{\left(V_{\max}-V_{\min}\right)}^{2}}{12}$, where $V_{min}$ and $V_{max}$ represent the minimum and maximum speed of vehicles, respectively.

**Assumption** **3** (Ideal channel conditions). *The vehicle nodes are under saturated conditions, which indicates that each node has WSA or RFS packets available to send after a successful reservation during the* WSA interval.

**Assumption** **4** (Ideal channel conditions). No bit errors due to channel fading, no hidden terminal problems and no capture effect, Thus, the packets transmission failure is only due to collisions.

**Assumption** **5** (Only one access category in one node). Each node only transmits a single non-safety traffic flow belonging to one AC, and thus, it avoids the need to take into account the interval virtual collisions [30].

We also assume that transmission rate on the CCH and SCHs are constant and the same. The non-safety messages on the SCHs have the same size, which means that all the non-safety messages occupy the same size of slot on the SCHs.

Now we analyze the minimum CW size for different service classes. There are four classes of service classified by different bandwidth requirement over SCHs corresponding to four ACs of WSA packets on the CCH. Different ACs in the IEEE 802.11p EDCA are identified by channel access parameter including CW and Arbitration Inter-frame Space (AIFS). The AIFS Number (AIFSN) is used to determine the duration of AIFS according to

(2) AIFS[i]=SIFS+AIFSN[i]×σ

where AIFSN[i] is an integer greater than or equal to 2, and SIFS and σ denote the duration of an Short Inter Frame Space (SIFS) and the duration of an idle time slot, respectively. Let $m^{\prime }$ and *m* denote the maximum times the CW can be doubled and the maximum number of retransmissions, respectively. For simplicity, we assume that each $AC_{i}$ (*i* = 0, 1, 2, 3) has the same *m* and $m^{\prime }$, and $AC_{0}$ and $AC_{3}$ corresponds to the highest priority and the lowest priority, respectively.

**Figure 2:**
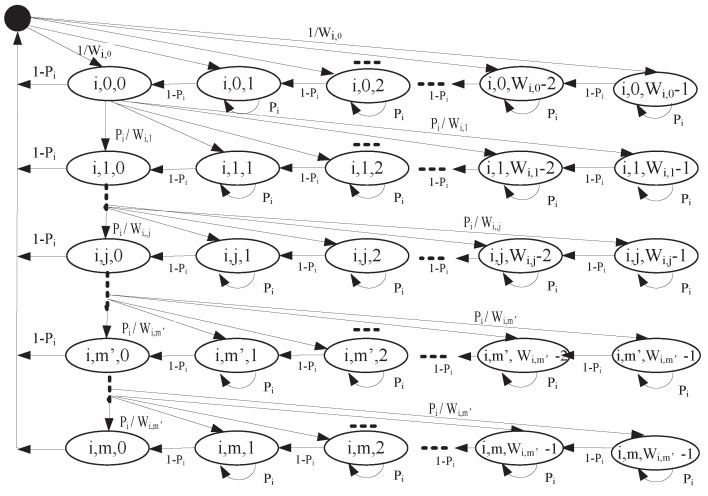
Two-dimensional Markov chain for the priority $AC_{i}$.

In order to derive the proper minimum CW size for different service classes, we model a two-dimensional Markov chain evolved from [15,31,32] to obtain the stationary probability $\tau_{i}$ that a node transmits a WSA or an RFS packet corresponding to $AC_{i}$ in an arbitrary time slot during *WSA interval*, as shown in Figure 2.

Let s(i,t) and b(i,t) be a stochastic process representing the backoff stage and backoff timer of WSA/RFS traffic belonging to $AC_{i}$ at time *t*, respectively. Let $W_{i,j}$ (j∈[0,m]) represent the CW in the *j*th backoff stage of $AC_{i}$. For the first transmission attempt, CW of $AC_{i}$ is set to the minimum value $W_{i,0}$. When the collision is detected, for the first $m^{\prime }$ steps, the CW of $AC_{i}$ is doubled and then retransmission is started. The CW remains unchanged for ($m-m^{\prime }$) steps. To sum up, the CW size of $AC_{i}$ in the *j*th stage, $W_{i,j}$, can be give as:

(3) $$\begin{array}{c} W_{i,j} \end{array}=\left\{\begin{array}{c} 2^{j}W_{i,0}\\ 2^{m^{\prime }}W_{i,0} \end{array}\right.\begin{array}{cc} & \begin{array}{c} \begin{array}{cc} j\leq & m^{\prime } \end{array}\\ \begin{array}{cc} m^{\prime }< & j \end{array}\leq m \end{array} \end{array}$$

Let $b_{i,j,k}=\lim_{t\to \infty }\left\{s(i,t)=j,b(i,t)=k\right\},0\leq j\leq m,0\leq k\leq W_{i,j}-1$ denote the stationary distribution of the chain of WSA/RFS traffic in Figure 2. Let $P_{i}$ denote the collision probability that a node delivering a WSA/RFS packet with $AC_{i}$ collides with other nodes when access the channel.

**Theorem** **1.** *The stationary probability $\tau_{i}$ that a vehicle node sends a WSA or an RFS packet corresponding to $AC_{i}$ in a random time slot is $\tau_{i}=\left(1-P_{i}^{m+1}\right)\cdot b_{i,0,0}/\left(1-P_{i}\right)$.*

**Proof.**  As shown in Figure 2, the one-step transition probabilities are:

(4) $$\left\{\begin{array}{c} P\left\{i,j,k|i,j,k\right\}=P_{i}\begin{array}{cc} & 0\leq k\leq W_{i,j}-1,0\leq j\leq m \end{array}\\ P\left\{i,j,k-1|i,j,k\right\}=1-P_{i}\begin{array}{cc} & 1\leq k\leq W_{i,j}-1,0\leq j\leq m \end{array}\\ P\left\{i,j,k|i,j-1,0\right\}\;=\;\left\{\begin{array}{c} \frac{P_{i}}{W_{i,j}}0\leq k\leq W_{i,j}-1,1\leq j\leq m^{\prime }\\ \frac{P_{i}}{W_{i,m^{\prime }}}0\leq k\leq W_{i,m^{\prime }}\;-\;1,m^{\prime }\;<\;j\leq m \end{array}\right.\\ P\left\{i,0,k|i,j,0\right\}\;=\;\left\{\begin{array}{c} \frac{\left(1-P_{i}\right)}{W_{i,0}}0\leq k\leq W_{i,0}-1,0\leq j\leq m-1\\ \frac{1}{W_{i,0}}\begin{array}{ccc} & & 0\leq k\leq W_{i,0}-1,j=m \end{array} \end{array}\right. \end{array}\right.$$

The meaning of each line in Equation (4) is as follows:

(1) When the channel is busy, the backoff timer is frozen;
(2) When the channel is free, the backoff timer will subtract one;
(3) Within $m^{\prime }$ backoff stage, collision makes backoff stage increase and CW double. Otherwise, collision makes CW remain $2^{m^{,}}W_{i,0}$;
(4) When a WSA/RFS packet is successfully transmitted or reaches its maximum retransmission number *m*, the backoff timer is reset.

From the Markov chain in the Figure 2, we can obtain that

(5) $$b_{i,j-1,0}\cdot P_{i}=b_{i,j,0}\begin{array}{ccc} \to & b_{i,j,0}={P_{i}}^{j}\cdot b_{i,0,0} & 1\leq j\leq m \end{array}$$

As the Markov chain is regular, we have

(6) $$\begin{array}{cc} b_{i,j,k} & =\frac{W_{i,j}-k}{W_{i,j}\left(1-P_{i}\right)}b_{i,j,0}\\ & =\left\{\begin{array}{c} \frac{2^{j}W_{i,0}-k}{2^{j}W_{i,0}\left(1-P_{i}\right)}b_{i,j,0},0\leq j\leq m^{\prime },1<k\leq W_{i,j}-1\\ \frac{2^{m^{\prime }}W_{i,0}-k}{2^{m^{\prime }}W_{i,0}\left(1-P_{i}\right)}b_{i,j,0},\begin{array}{c} m^{\prime }<j\leq m,1<k\leq W_{i,j}-1. \end{array} \end{array}\right. \end{array}$$

Thus, applying the normalization condition for stationary distribution, we can have

(7) $$\begin{array}{c} 1=\sum_{j=0}^{m}\sum_{k=0}^{W_{i,j}-1}b{}_{i,j,k}\\ \;=\sum_{j=0}^{m}b_{i,0,0}P_{i}^{j}+\sum_{j=0}^{m}\sum_{k=1}^{W_{i,j}-1}\frac{W_{i,j}-k}{W_{i,j}\left(1-P_{i}\right)}b_{i,j,0} \end{array}$$

Using Equations (5)–(7), we get

(8) $$\frac{1}{b_{i,0,0}}=\left\{\begin{array}{c} \frac{1}{2(1-P_{i})}\left[\frac{\left[1-{\left(2P_{i}\right)}^{m+1}\right]W_{i,0}}{1-2P_{i}}-\frac{1-P_{i}^{m+1}}{1-P_{i}}\right]\\ +\frac{1-P_{i}^{m+1}}{1-P_{i}}\begin{array}{cc} , & m\leq m^{\prime } \end{array}\\ \frac{1}{2(1-P_{i})}[\frac{2^{m^{\prime }}W_{i,0}\left(P_{i}^{m^{\prime }+1}-P_{i}^{m+1}\right)-1+P_{i}^{m+1}}{1-P_{i}}\\ +\frac{\left[1-{\left(2P_{i}\right)}^{m^{\prime }+1}\right]W_{i,0}}{1-2P_{i}}]+\frac{1-P_{i}^{m+1}}{1-P_{i}}\begin{array}{cc} , & m>m^{\prime }. \end{array} \end{array}\right.$$

Then, the transmission probability $\tau_{i}$ can be expressed as

(9) $$\tau_{i}=\sum_{j=0}^{m}b_{i,j,0}=\sum_{j=0}^{m}P_{i}^{j}\cdot b_{i,0,0}=\frac{1-P_{i}^{m+1}}{1-P_{i}}b_{i,0,0}.$$

where $b_{i,0,0}$ is shown in Equation (8).

Let $N_{i}$ denote the number of nodes delivering non-safety packets which belong to $AC_{i}$. The total number of nodes, *N*, can be expressed as

(10) $$N=\sum_{i=0}^{3}N_{i}$$

Collision occurs when more than one node are transmitting at the same time slot, so the collision probability $P_{i}$ is

(11) $$P_{i}=1-{\left(1-\tau_{i}\right)}^{N_{i}-1}\prod_{h=0,h\neq i}^{3}{\left(1-\tau_{h}\right)}^{N_{h}}$$

According to Equations (9) and (11), variables $\tau_{i}$ and $P_{i}$ can be solved by the numerical methods in [32]. Note that $0<\tau_{i}<1$ and $0<P_{i}<1$. ☐

Let $P_{i,suc}$ denote the probability that a node transmits WSA/RFS packets belonging to $AC_{i}$ in a slot time to make a successful reservation during the *WSA interval* on the CCH, and thus, $P_{i,suc}$ can be computed as follows:

(12) $$P_{i,suc}=N_{i}\tau_{i}{\left(1-\tau_{i}\right)}^{N_{i}-1}\prod_{h=0,h\neq i}^{3}{\left(1-\tau_{h}\right)}^{N_{h}}$$

By following the procedure in [31], the saturation throughput $S_{i,cch}$ for the traffic belonging to $AC_{i}\left(i=1,2,3,4\right)$ on CCH can be calculated by

(13) $$S_{i,cch}=\frac{P_{i,suc}L_{wsa}}{\left(1-P_{b}\right)\sigma +\sum_{i=0}^{3}P_{i,suc}\left(T_{s}+AIFS\left[i\right]\right)+\left(P_{b}-\sum_{i=0}^{3}P_{i,suc}\right)T_{col}}$$

where $P_{b}$, $T_{s}$, $T_{col}$ and $L_{wsa}$ represent the probability that the channel is busy (i.e, at least one node transmits during a time slot), the average time used for successfully transmitting a WSA packet (It does not include AIFS[i]), the average time wasted by a WSA packet collision and the average payload of a WSA packet, respectively. $P_{b}$ can be calculated by

(14) $$P_{b}=1-\prod_{h=0}^{3}{\left(1-\tau_{h}\right)}^{N_{h}}$$

We assume that the $L_{wsa}$ for different traffic belonging to $AC_{i}\;\left(i=1,\;2,\;3,\;4\right)$ is equal. $T_{col}$ can be calculated according to Equation (21). Now we analyze the throughput obtained according to Equation (13). It is clear that, for different traffic belonging to $AC_{i}\left(i=1,\;2,\;3,\;4\right)$, the denominator of Equation (13) is the same, and the $L_{wsa}$ is also equal as assumed. Therefore, the throughput $S_{i,cch}$ can be represented by $P_{i,suc}$. Let $S_{i}$ denote the throughput of nodes delivering non-safety packets belonging to $AC_{i}$ on SCHs. The throughput $S_{i}$ obtained on SCHs is determined by the number of successful SCH reservations of nodes with $AC_{i}$ on CCH, and is ultimately determined by the throughput, $S_{i,cch}$, on CCH. Therefore, the $S_{i}$ can be represented by $P_{i,suc}$.

In order to offer QoS supported delivery in SCHs, different ACs have different minimum CWs. Let $S_{j}$ and $S_{g}$ (j,g=0,1,2,3) be the saturation throughput obtained on SCHs due to delivering service data packets belonging to $AC_{j}$ and $AC_{g}$, respectively. Thus, we have

(15) $$\begin{array}{cc} R_{S_{j}/S_{g}} & =\frac{S_{j}}{S_{g}}=\frac{P_{j,suc}}{P_{g,suc}}\\ & =\frac{N_{j}\tau_{j}{\left(1-\tau_{j}\right)}^{N_{j}-1}\prod_{h=0,h\neq j}^{3}{\left(1-\tau_{h}\right)}^{N_{h}}}{N_{g}\tau_{g}{\left(1-\tau_{g}\right)}^{N_{g}-1}\prod_{h=0,h\neq g}^{3}{\left(1-\tau_{h}\right)}^{N_{h}}}\\ & =\frac{N_{j}\tau_{j}\left(1-\tau_{g}\right)}{N_{g}\tau_{g}\left(1-\tau_{j}\right)} \end{array}$$

where $R_{S_{j}/S_{g}}$ represents the ratio of $S_{j}$ to $S_{g}$. If the predefined throughput ratio between different service classes and the minimum CW for any certain non-safety service class is given, we can obtain the minimum CWs for other service classes.

## 4. Performance Analysis

In this section, we analyze the throughput and delay of our proposed model in a highway environment.

### 4.1. Throughput Analysis

Note that, the throughput obtained according to Equation (13) is based on a single channel environment, and it does not consider the features of channel switching defined in IEEE 1609.4 protocol [10]. Although throughput $S_{i}$ is determined by $P_{i,suc}$ in both single channel and channel switching environments, we still need another way to obtain throughput $S_{i}$ under multichannel environments.

We use a contention model to analyze the average time consumed on CCH for a SCH reservation. In each time slot during the *WSA interval*, channel is idle with probability $P_{idle}$. Otherwise, the transmitting is successful with probability $P_{suc}$ and channel collision occurs with probability $P_{col}$.

(16) $$P_{idle}=\prod_{h=0}^{3}{\left(1-\tau_{h}\right)}^{N_{h}}$$

(17) $$\begin{array}{cc} P_{suc} & =\sum_{i=0}^{3}P_{i,suc}\\ \; & =\sum_{i=0}^{3}\left\{N_{i}\tau_{i}{\left(1-\tau_{i}\right)}^{N_{i}-1}\prod_{h=0,h\neq i}^{3}{\left(1-\tau_{h}\right)}^{N_{h}}\right\} \end{array}$$

(18) $$\begin{array}{cc} P_{col} & =1-P_{idle}-P_{suc}\\ & =1-\prod_{h=0}^{3}{\left(1-\tau_{h}\right)}^{N_{h}}\\ & \;-\sum_{i=0}^{3}\left\{N_{i}\tau_{i}{\left(1-\tau_{i}\right)}^{N_{i}-1}\prod_{h=0,h\neq i}^{3}{\left(1-\tau_{h}\right)}^{N_{h}}\right\} \end{array}$$

Let $T_{reser}$ denote the duration from the time instant when a WSA or an RFS becomes the head of the MAC to the time instant when a reservation is successfully made or dropped due to reaching the maximum retransmission number. We assume that the nodes are under saturated conditions. Let *Z* represent the time interval between two consecutive free time slots before a reservation is successfully made. According to the backoff mechanism, the probability of consecutive packets collisions or consecutive successful reservations is too small to be considered [15,33]. So, variable *Z* only contains a collision or a free time slot, therefore, we can have

(19) Z=σ+B

the random variable *B* can be expressed as follows:

(20) $$B=\left\{\begin{array}{c} 0,P_{idle}/\left(P_{idle}+P_{col}\right)\\ T_{col},\begin{array}{cc} & P_{col}/\left(P_{idle}+P_{col}\right). \end{array} \end{array}\right.$$

where $T_{col}$ denotes the time of a collision, and is composed of a maximum AIFS time, AIFS[∗], the time $T_{wsa}$ for transmitting a WSA/RFS packet, and the duration of the propagation delay δ. For simplicity, we set $T_{wsa}$ equal to the time $T_{rfs}$ for transmitting an RFS, i.e., $T_{wsa}=T_{rfs}$. Then, we have

(21) $$\left\{\begin{array}{cc} T_{col} & =AIFS\left[*\right]+T_{wsa}+\delta \\ T_{wsa} & =\frac{L_{wsa}}{R_{d}} \end{array}\right.$$

where $R_{d}$ represents the data rate on both CCH and SCHs.

According to Equations (19) and (20), the mean interval of variable *Z* can be expressed as,

(22) $$E\left[Z\right]=\sigma +\frac{P_{col}\cdot T_{col}}{P_{idle}+P_{col}}$$

The probability that successful transmission occurs after the *k*th free time slot follows a geometric distribution.

(23) $$P\left(K=k\right)={\left(1-P{}_{suc}\right)}^{k-1}\cdot P_{suc},\begin{array}{cc} & k=1,2,3\ldots \end{array}$$

Based on Equations (22) and (23), the mean reservation time can be given by:

(24) $$\begin{array}{cc} E\left[T_{reser}\right] & =\left(\frac{1}{P{}_{suc}}-1\right)\cdot E\left[Z\right]+\sigma +T_{suc}\\ & =\frac{\sigma +P_{col}\cdot T{}_{col}}{P_{suc}}+T_{suc}. \end{array}$$

where $T_{suc}$ denotes the duration of a successful reservation. Let $T_{ack}=L_{ack}/R_{d}$ be the cost of transmitting the payload $L_{ack}$ of an ACK packet. Since ACs with longer AIFS are prevented from accessing some channel [4,34], we also consider the different AIFS for different ACs, $T_{suc}$ can be calculated by

(25) $$\begin{array}{cc} T_{suc} & =\frac{\sum_{i=0}^{3}P_{i,suc}(AIFS\left[i\right]+T_{wsa}+SIFS+T_{ack}+2\delta )}{P_{suc}}\\ & =\frac{\sum_{i=0}^{3}P_{i,suc}(AIFS\left[i\right]+T_{wsa}+SIFS+T_{ack}+2\delta )}{\sum_{i=0}^{3}P_{i,suc}} \end{array}$$

To calculate the throughput of SCHs, we define the following symbols.

(1) Let $T_{SI}$ and *M* denote the length of a SI and the number of lanes in each direction, respectively.
  The RSU estimates the number of newly entering vehicles, $n_{new}$, during a SI by [22]
  (26) $$n_{new}=2M\cdot V_{avg}\cdot \beta \cdot T_{SI}$$
  $T_{VII}$ can be estimated as follows [22]:
  (27) $$T_{VII}=L_{total}\cdot T_{rrts}+m^{\prime \prime }\cdot T_{cp}$$
  where $T_{cp}$ and $T_{rrts}$ represent the duration of transmitting a Coordination Packet (CP) and an Reservation Request To Send (RRTS) packet, respectively. $m^{\prime \prime }$ and $L_{total}$ in Equation (27) represent the number of rounds that a node has to be experienced before it is identified by RSU and the total Frame length, respectively which can be easily derived according to Algorithm 1 in [22].
(2) Let $T_{SaSlot}$ denote the duration for transmitting a safety-related message. According to Figure 1, we have
  (28) $$T_{SI}=T_{CFI}+T_{VII}+T_{WI}$$
  (29) $$T_{CFI}=N\cdot T_{SaSlot}+2T_{CLI}.$$
  where $T_{CLI}$ denotes the transmission delay of a CLI packet, and now, we calculate it. As an example of large dimensioning, we assume that the maximum number of nodes which can exist in an RSU converge is $N_{max}$, and $N_{max}$ equals to 200. A CLI packet includes scheduling slot information of each identified node during CFI, the minimum CW size for four ACs, $T_{CFI}$, $T_{VII}$ and $T_{WI}$. If the maximum number of nodes is $N_{max}$, at least $\left\lceil log_{2}N_{max}\right\rceil$ bits are required to represent a node ID, where ⌈·⌉ denotes the ceiling function. An ID of 8 bits is sufficient for the network size assumed. Since each node corresponds to a safety slot during CFI, 8 bits are sufficient to identify a safety slot. Since the number of different ACs, $N_{AC}$, is four and the maximum CW, $CW_{max}$, is 1024 in IEEE 802.11p [9] and IEEE 1609.4 protocol [10], and thus 2 bits and 10 bits are sufficient to represent a AC and a minimum CW, respectively. Due to an SI with a length of 100 ms, 8 bits are sufficient to represent, respectively, $T_{CFI}$, $T_{VII}$ and $T_{WI}$. Therefore, we can derive $T_{CLI}$ by
  (30) $$\begin{array}{cc} T_{CLI}= & \frac{2\left\lceil \log_{2}N_{\max}\right\rceil N_{\max}}{R_{d}}\\ \; & +\frac{\left(\left\lceil \log_{2}N_{AC}\right\rceil +\left\lceil \log_{2}CW_{\max}\right\rceil \right)N_{AC}}{R_{d}}\\ \; & +\frac{\left\lceil \log_{2}T_{CFI}\right\rceil +\left\lceil \log_{2}T_{VII}\right\rceil +\left\lceil \log_{2}T_{WI}\right\rceil }{R_{d}}\\ = & \frac{\left[2\times 8\times 200+(2+10)\times 4+3\times 8\right]bits}{6Mbps}\\ = & 0.55\;\mathrm{ms} \end{array}$$
  Therefore, we let $T_{CLI}$ be 1 ms in this paper.
(3) Let $N_{sch}$ represent the number of available SCHs in the VANETs.
(4) Let $G_{cch}$ denote the total number of successful SCHs reservation on CCH during the *WSA interval*. Let $G_{sch}$ be the number of non-safety packets transmitted over all $N_{sch}$ SCHs during the whole SI. We have
  (31) $$G_{cch}=\frac{T_{WI}}{E[T_{reser}]}.$$
  (32) $$G_{sch}=\frac{N_{sch}\cdot T_{SI}}{T_{data}}$$
  where $T_{data}$ denotes the duration of a non-safety packet transmission on the SCH. Therefore, $T_{data}$ can be calculated by
  (33) $$T_{data}=DIFS+T_{h}+T_{da}+SIFS+T_{ack}+2\delta$$
  where DIFS is the cost of a Distributed Inter Frame Space (DIFS), $T_{h}$ is the cost of MAC header and PHY header attached to the non-safety packet, and $T_{da}=L_{data}/R_{d}$ denotes the duration of transmitting the payload $L_{data}$ of a non-safety packet. Note, we still use constant DIFS instead of AIFS for clarify and simplicity when calculate $T_{data}$ for non-safety packets with different ACs.

The real number of non-safety packets on all SCHs satisfies such situation that the number of reservations made on CCH during *WSA interval* equals to the number of non-safety packets transmitted on all SCHs during SI, i.e., $G_{cch}=G_{sch}$. This means that, there are not enough idle time slots left in the *WSA interval* for making more reservations on CCH or in the SI for transmitting more non-safety packets on all SCHs. Therefore, the total throughput obtained on SCHs, $S_{sch}$, can be given by

(34) $$S_{sch}=\frac{\min\left(\frac{T_{WI}}{E[T_{reser}]},\frac{N_{sch}\cdot T_{SI}}{T_{data}}\right)\cdot L_{data}}{T_{SI}}$$

Without loss of generality, we consider two priority classes, let’s say $AC_{0}$ and $AC_{1}$. Given the predefined throughput ratio $R_{S_{0}/S_{1}}$ of non-safety service with $AC_{0}$ to $AC_{1}$, then the throughput obtained on SCHs of nodes delivering non-safety packets with $AC_{0}$ and $AC_{1}$, respectively, $S_{0}$ and $S_{1}$, can be calculated by

(35) $$\left\{\begin{array}{c} S_{0}=S_{sch}\times \frac{R_{S_{0}/S_{1}}}{1+R_{S_{0}/S_{1}}}\\ S_{1}=S_{sch}\times \frac{1}{1+R_{S_{0}/S_{1}}} \end{array}\right.$$

### 4.2. Delay Analysis

The delay of the non-safety message transmissions is the interval from the time instant that a non-safety packet contends to access the CCH to the time instant that this packet is successfully transmitted on SCH. The delay includes three parts: *WSA interval* (during which nodes perform SCH reservations), SI (during which the non-safety packets are transmitted) and synchronization intervals (which the nodes need to be experienced before a successful transmission). As the SCH reservations and the transmissions of non-safety messages are random, the delay in the *WSA interval* and SI can be approximated as half of each interval length.

The main factor of delay is the number of synchronization intervals before a successful transmission due to relatively long value compared to the other two parts. If a node, due to unsuccessful reservation during the *WSA interval* or without enough time for transmission on SCH during the next SI, does not successfully perform a transmission, it will wait for the next next SI and go on until a successful transmission. Let $\zeta_{0}$ and $\zeta_{1}$ be the average number of successful transmissions on the SCHs of each node with $AC_{0}$ and $AC_{1}$ in an synchronization interval, respectively. It can be calculated by the total number of successful transmissions during an synchronization interval over the total number of contending nodes, as given by,

(36) $$\left\{\begin{array}{c} \zeta_{0}=\frac{n_{0,suc}}{N_{0}}\\ \zeta_{1}=\frac{n_{1,suc}}{N_{1}} \end{array}\right.$$

where $n_{0,suc}$ and $n_{1,suc}$ denote the number of successful transmissions of non-safety packets with $AC_{0}$ and $AC_{1}$ on the SCHs during an synchronization interval, respectively, and they can be calculated by

(37) $$\left\{\begin{array}{c} n_{0,suc}=\min\left(\frac{T_{WI}}{E[T_{reser}]},\frac{N_{sch}\cdot T_{SI}}{T_{data}}\right)\times \frac{R_{s_{0}/s_{1}}}{1+R_{s_{0}/s_{1}}}\\ n_{1,suc}=\min\left(\frac{T_{WI}}{E[T_{reser}]},\frac{N_{sch}\cdot T_{SI}}{T_{data}}\right)\times \frac{1}{1+R_{s_{0}/s_{1}}} \end{array}\right.$$

Therefore, the total transmission delay $T_{0,delay}$ and $T_{1,delay}$ of non-safety packets with $AC_{0}$ and $AC_{1}$, respectively, can be expressed as

(38) $$\left\{\begin{array}{cc} T_{0,delay} & =\frac{1}{2}\cdot T_{WI}+\left(\frac{1}{\zeta_{0}}-1\right)\cdot T_{SI}+\frac{1}{2}\cdot T_{SI}\\ & =\frac{1}{2}\cdot T_{WI}+\left(\frac{1}{\zeta_{0}}-\frac{1}{2}\right)\cdot T_{SI}\\ T_{1,delay} & =\frac{1}{2}\cdot T_{WI}+\left(\frac{1}{\zeta_{1}}-1\right)\cdot T_{SI}+\frac{1}{2}\cdot T_{SI}\\ & =\frac{1}{2}\cdot T_{WI}+\left(\frac{1}{\zeta_{1}}-\frac{1}{2}\right)\cdot T_{SI} \end{array}\right.$$

## 5. Performance Evaluation

In this section, we evaluate the performance of the proposed EQM-MAC protocol through a simulation study. We compare the performance of EQM-MAC protocol with the following protocols (schemes).

- The IEEE 1609.4 protocol [10]: This is the default multichannel protocol with fixed CCH interval (50 ms) and SCH interval (50 ms). All nodes use the CSMA/CA mechanism to perform channel access for the transmissions of safety-related and WSA messages on the CCH, and switch to the specific SCH to disseminate non-safety messages during the SCHI.
- The multichannel TDMA MAC protocol specifically for VANETs scenario (VeMAC) [17]: VeMAC protocol is considered to be the very beginning in research of TDMA MAC for V2V communication. Each node has two transceivers: Transceiver 1 is always tuned to CCH to transmit safety messages and make SCH reservations while transceiver 2 can be tuned to any SCH to transmit non-safety messages. The VeMAC protocol works in a distributed way, and thus each node requires to exchange additional information to obtain a time slot for transmitting safety-related messages [17]. According to the protocol, the length of a VeMAC (packet) is about 650 bytes ($N_{max}=200$), and the duration for transmitting this packet is thus about 0.9 ms given $R_{d}$ = 6 Mbps [17]. In the following analysis, the length of each frame defined in VeMAC protocol is 200. For facilitation of the analysis, each node always makes a successful SCH reservation in a frame, and service provider can only transmit one service packet for a successful SCH reservation in a frame.
- The Coordinated multichannel MAC (C-MAC) protocol [16]: With the coordination of the RSU, C-MAC protocol provides contention-free broadcasting for safety-related messages, and thus lowers the collision probabilities for transmissions of safety-related messages. Through optimizing the SCH interval, the maximal saturation throughput of SCHs is obtained.
- The QoS supported Variable CCH Interval (Q-VCI) MAC protocol [5]: The Q-VCI protocol can support the QoS delivery in a multi-rate multichannel VANETs environments by adjusting the minimum CW for different service classes at each node. In addition, the Q-VCI protocol adaptively tunes the CCHI to ensure the transmissions of safety-related messages and to maximize the throughput of SCHs according to the traffic conditions. We set α, data rate for two SCHs and data rate for the other SCHs, respectively, be 3, 6 Mbps and 9 Mbps in the Q-VCI MAC protocol.

### 5.1. Simulation Scenario

**Figure 3:**
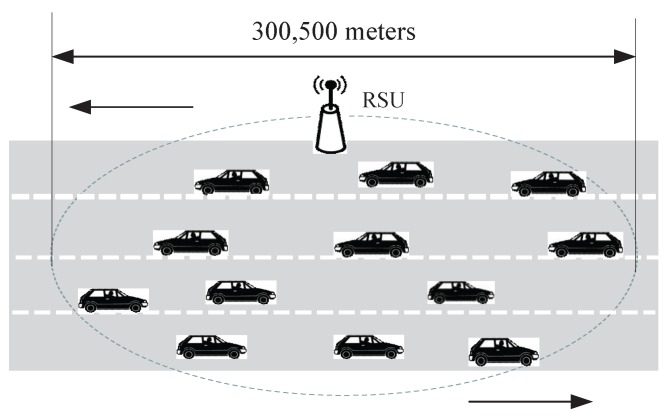
A scenario with 2 lanes in each direction on highway with RSU and moving vehicles.

The simulation platform is the network simulator NS3 [35], in which V2V and V2I communicate over an experience Rayleigh fading channel. The simulation scenario is on a 6-km-long highway with 2-lanes in each direction as shown in Figure 3. The speed of vehicles is uniformly distributed in [80, 120] km/h and [60, 100] km/h. We set the duration $T_{SaSlot}$ of each slot in the CFI be 0.4 ms when safety-related packet size is 200 bytes and transmission data rate is 6 Mbps. Every vehicle has a GPS and a single-radio WAVE communication device. All nodes can act as both service providers and service users. Simulation time is 2 min and the final result is the average of each simulation result. We evaluate our proposed EQM-MAC protocol under different traffic densities to guarantee scalability, reliability and efficiency. Configuration parameters are summarized in Table 3.

### 5.2. Simulation Results

**Figure 4:**
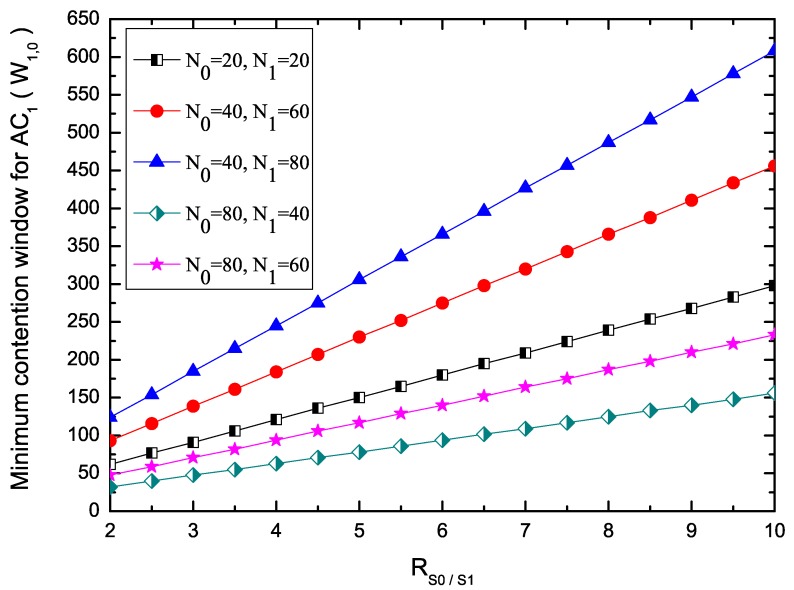
Minimum contention window for $AC_{1}$.

Figure 4 shows the minimum Contention Window (CW) for $AC_{1}$ under different $R_{S_{0}/S_{1}}$. The minimum CW for $AC_{0}$ is 32. When the number of nodes delivering each AC packets is fixed, the minimum CW for nodes belonging to $AC_{1}$, $W_{1,0}$, increases with higher $R_{S_{0}/S_{1}}$. This is because that, when the number of nodes is fixed, the larger CW incurs smaller probabilities of successful reservations, and thus lower throughput can be achieved on the SCHs, which can be seen from Equation (15). It is evident that the minimum CW for nodes with $AC_{1}$ become larger when fewer nodes with $AC_{0}$ deliver packets. For example, if $R_{S_{0}/S_{1}}$ = 4, $W_{1,0}$ = 184 when $N_{0}$ = 40 and $N_{1}$ = 60, while $W_{1,0}$ = 245 when $N_{0}$ = 40 and $N_{1}$ = 80. This is because, based on predefined value ($R_{S_{0}/S_{1}}$ and $W_{0,0}$), EQM-MAC protocol can adjust minimum CW for other service classes to ensure the packets with higher priority to be transmitted. Therefore, the EQM-MAC protocol can differentiate the transmission opportunities for the packets with different service classes.

**Figure 5:**
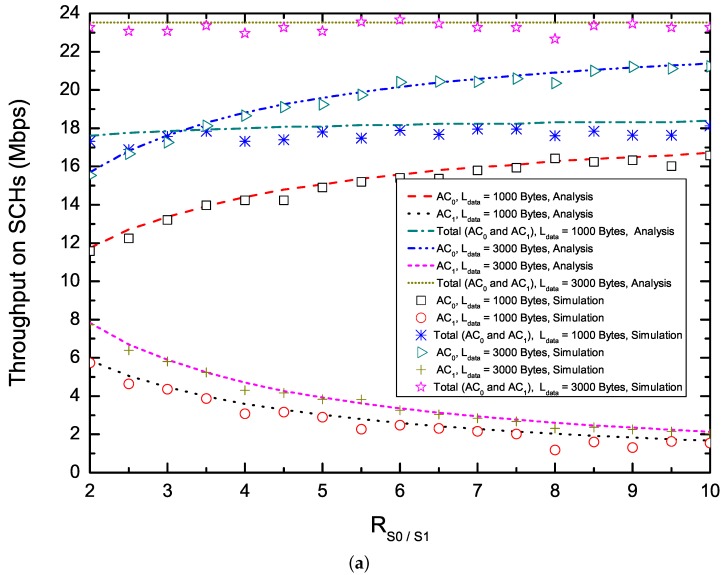

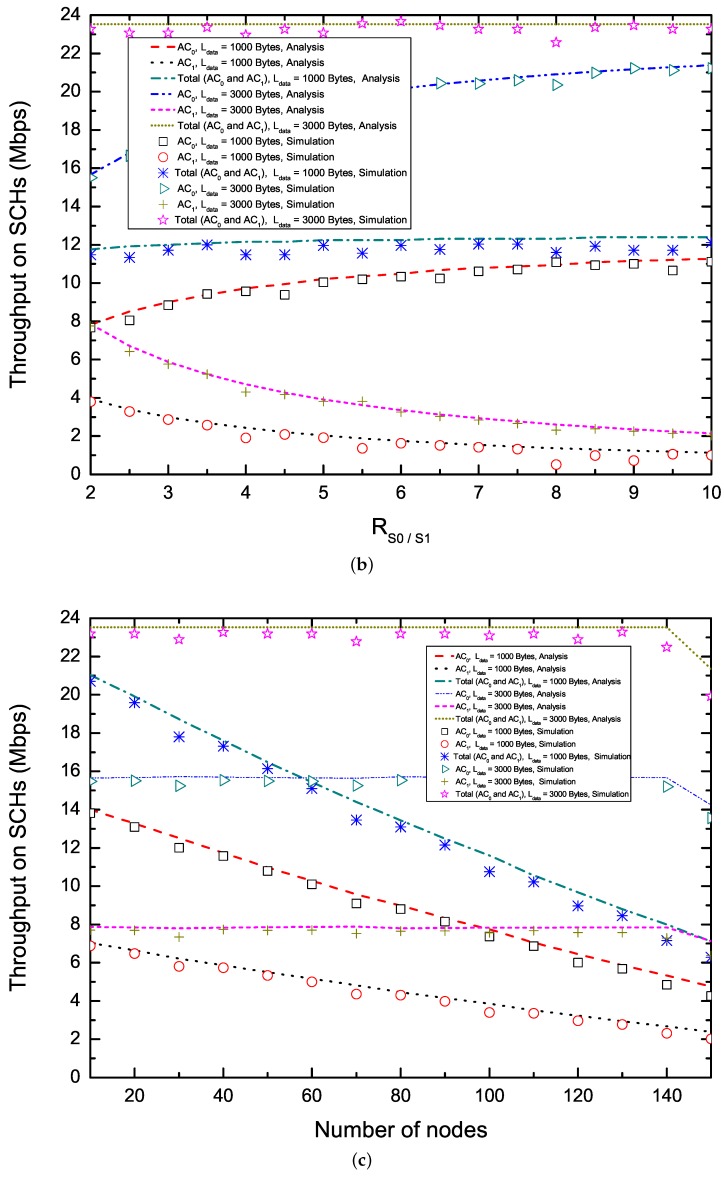

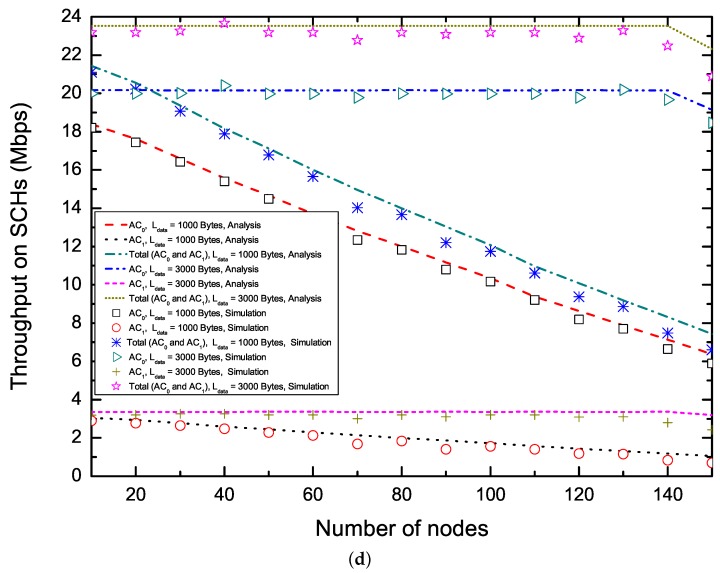
Saturation throughput on SCHs. (**a**) Saturation throughput versus $R_{S_{0}/S_{1}}$ ($N_{0}$ = 20, $N_{1}$ = 20);(**b**) Saturation throughput versus $R_{S_{0}/S_{1}}$ ($N_{0}$ = 40, $N_{1}$ = 60); (**c**) Saturation throughput versus the number of nodes ($N_{0}$ = $N_{1}$, $R_{S_{0}/S_{1}}$ = 2); (**d**) Saturation throughput versus the number of nodes ($N_{0}$ = $N_{1}$, $R_{S_{0}/S_{1}}$ = 6.)

Figure 5 shows the saturation throughput on SCHs in terms of $R_{S_{0}/S_{1}}$ (Figure 5a,b) and the number of nodes delivering two ACs (Figure 5c,d). To enhance the utilization of SCHs, EQM-MAC protocol allows each node to make reservations and transmit non-safety packets multiple times on SCHs during an synchronization interval. From Figure 5a,b, we can observe that, with the increase of $R_{S_{0}/S_{1}}$, the saturation throughput for $AC_{0}$ increases, while the saturation throughput for $AC_{1}$ decreases. This is because that, when the number of nodes delivering two ACs is fixed, with the increase of $R_{S_{0}/S_{1}}$, the proposed EQM-MAC protocol allocates larger CW to nodes delivering packets with $AC_{1}$, and thus the saturation throughput for $AC_{0}$ increases and the saturation throughput for $AC_{0}$ decreases. From Figure 5c,d, we find that, the total throughput for packets with payload $L_{data}$ = 3000 bytes keeps its maximum level at first and then reduces when the number of nodes rises further, while the throughput for packets with payload $L_{data}$ = 1000 bytes decreases with the increase of the number of nodes. The reasons is that, taking $L_{data}$ = 3000 bytes and $R_{S_{0}/S_{1}}$ = 2 for example, when the number of nodes is less than 140, each node has a great chance to make SCH reservations and has enough time to transmit on SCHs. However, when the number of nodes becomes larger, due to the increase of $T_{reser}$ and the shortage of the *WSA interval*, each node has little chance to make SCH reservations and thus the probability of successful reservations decreases. Since each transmission with long payload $L_{data}$ carries more data than that of short payload $L_{data}$, total throughput of long payload $L_{data}$ is higher than that of short payload $L_{data}$. It is also clear that the throughput of packets with $AC_{0}$ is more than that of packets with $AC_{1}$ as shown in Figure 5, which demonstrates the QoS differentiation in the EQM-MAC protocol. Analytical and simulation results match well. Our proposed protocol is validated.

**Figure 6:**
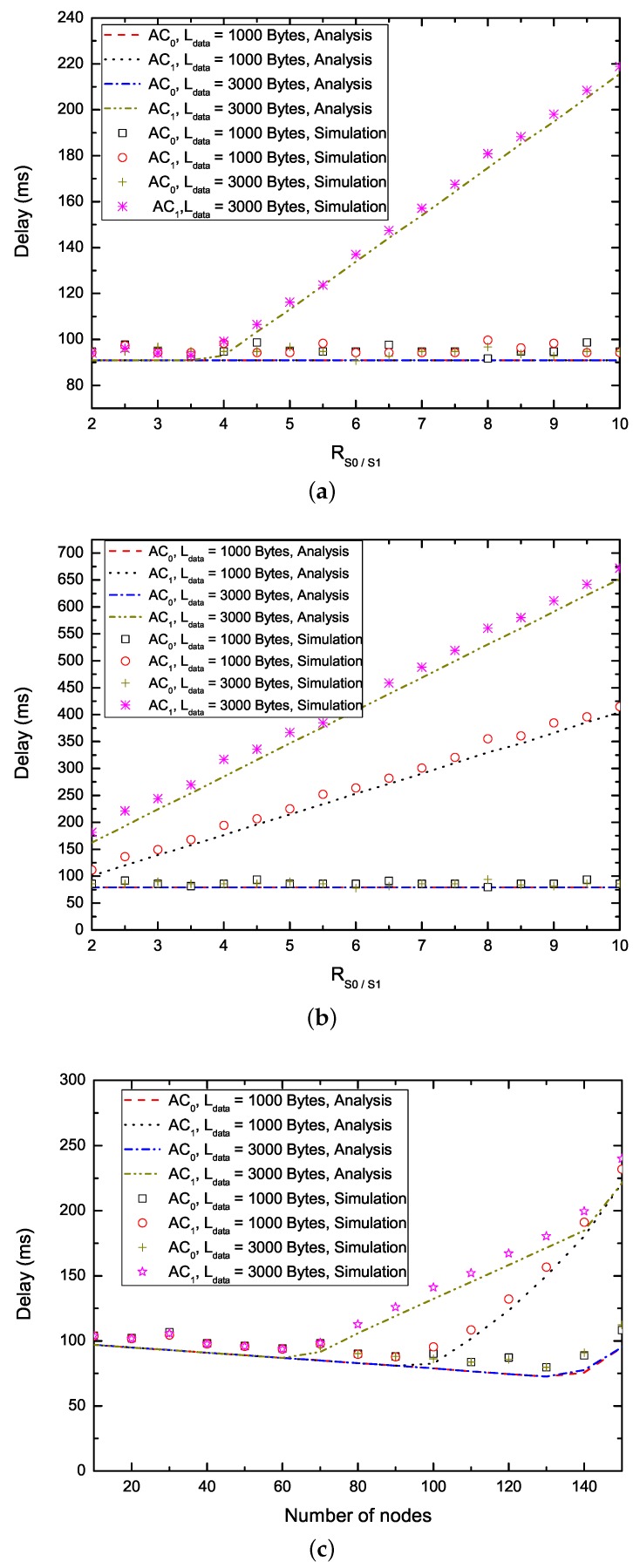

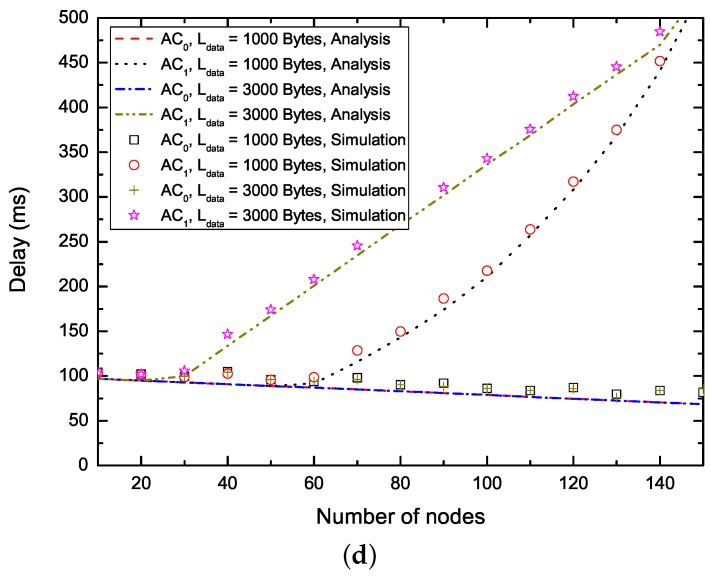
Non-safety packet delay. (**a**) Delay versus $R_{S_{0}/S_{1}}$ ($N_{0}$ = 20, $N_{1}$ = 20); (**b**) Delay versus $R_{S_{0}/S_{1}}$ ($N_{0}$ = 40, $N_{1}$ = 60); (**c**) Delay versus the number of nodes ($N_{0}$ = $N_{1}$, $R_{S_{0}/S_{1}}$ = 2); (**d**) Delay versus the number of nodes ($N_{0}$ = $N_{1}$, $R_{S_{0}/S_{1}}$ = 6.)

Figure 6 shows the analysis and simulation results of the delay performance of the proposed EQM-MAC protocol on the basis of $R_{S_{0}/S_{1}}$ (Figure 6a,b) and the number of nodes delivering two ACs (Figure 6c,d). From Figure 6a,b, we can see that, on one hand, with the increase of $R_{S_{0}/S_{1}}$, the packets with $AC_{0}$ maintain the same lower delay. Therefore, the proposed EQM-MAC protocol can ensure the near-real time needs of some interactive entertainment applications such as mobile multiplayer gaming and voice over IP. On the other hand, with the increase of $R_{S_{0}/S_{1}}$, the delay for packets with $AC_{1}$ rises. The reason is that, when the number of nodes delivering packets belonging to $AC_{0}$ ($AC_{1}$) is fixed, with the increase of $R_{S_{0}/S_{1}}$, the EQM-MAC protocol allocates larger CW to nodes delivering packets with $AC_{1}$ to ensure the reservation of higher priority AC, which reduces the probability of successful reservations for nodes delivering $AC_{1}$, and thus the higher priority AC has a higher probability of successful reservations and maintains the same lower delay. Therefore, the EQM-MAC protocol has the capability of QoS differentiation. In addition, different payloads have no effect on transmission delay of packets with $AC_{0}$, and they have the same delay. The reason is that, EQM-MAC protocol can give larger CW for nodes with $AC_{1}$ to guarantee the reservation of higher priority AC ($AC_{0}$), and thus the nodes with $AC_{0}$ (although have different payloads) all have great chances to make SCH reservations and have enough time to transmit on SCHs. The delay decreases first and then increases when the number of nodes becomes larger, as shown in Figure 6c,d. Since the number of nodes increases, the increase of *safety interval* incurs a shorter *WSA interval*. We take the nodes delivering packets with $AC_{1}$ and $L_{data}$ = 1000 bytes in Figure 6c for example to perform the following analysis. When the number of nodes is below 100, the length $T_{WI}$ of *WSA interval* accounts for the major part of the delay. Moreover, each node can make reservations at least once and all reservations can be successfully transmitted on SCHs. Therefore, the delay decreases to less than 100 ms. As the number of nodes further increases, it leads to a shorter *WSA interval*, and thus more intense contentions and less reservations. The nodes need more than one synchronization interval to transmit and thus the delay increases. When the number of nodes is 100, for example, the number of successful reservations of WSA packets is still more than the number of nodes. According to Equations (36) and (38), the delay is thus less than 100 ms. From Figure 6c, we also can observe that different values of payload $L_{data}$ or different ACs can bring about the same value of delay. For example, the delay of all nodes delivering two ACs is the same in the range of 10 nodes and 60 nodes. This is because each nodes cana successfully make reservations at least once and successfully transmit on SCHs at the arrival of the next next synchronization interval. When the number of nodes delivering the packets with $AC_{1}$ is in the range of 70 and 130, the delay of transmissions with long payload ($L_{data}$ = 3000 bytes) is larger than that with short payload ($L_{data}$ = 1000 bytes). This is because the number of successful reservations is the same for various payloads, but the transmission with longer payload incurs fewer available slots on SCHs and requires more synchronization intervals. When the number of nodes reaches 140, the transmissions of packets belonging to $AC_{1}$ with all kinds of payload have the same delay. This is due to the fact that the number of successful reservations is smaller and less than the number of nodes, so the nodes require several synchronization intervals before making successful reservations. Again, the analytical results match the simulation curves very well.

**Figure 7:**
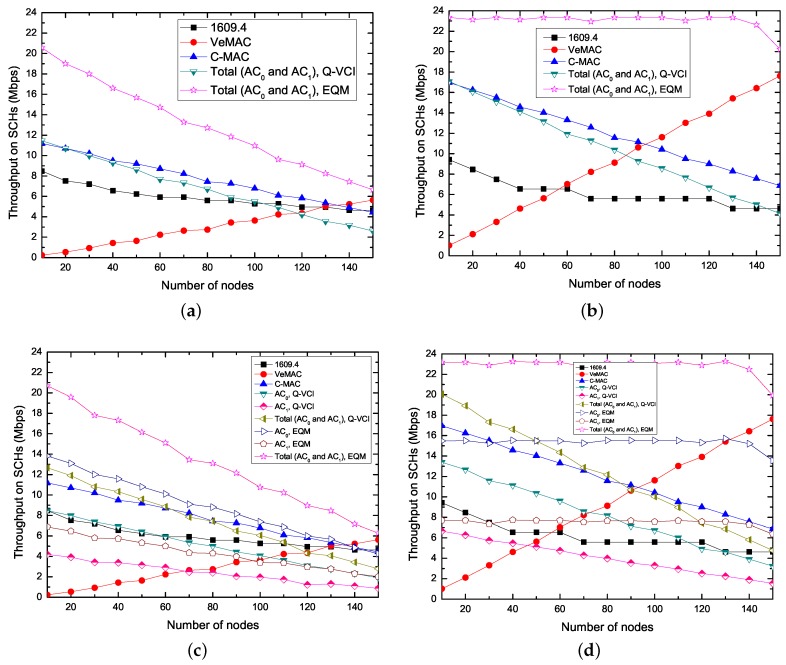

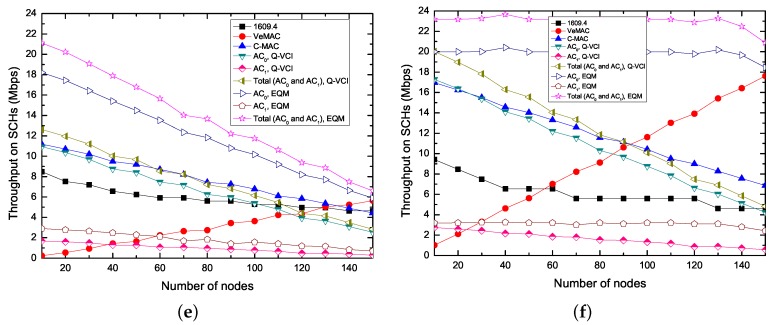
Saturation throughput on SCHs versus the number of nodes for kinds of protocols ($N_{0}$ = $N_{1}$). (**a**) $R_{S_{0}/S_{1}}$ = 1, $L_{data}$ = 1000 bytes; (**b**) $R_{S_{0}/S_{1}}$ = 1, $L_{data}$ = 3000 bytes; (**c**) $R_{S_{0}/S_{1}}$ = 2, $L_{data}$ = 1000 bytes; (**d**) $R_{S_{0}/S_{1}}$ = 2, $L_{data}$ = 3000 bytes; (**e**) $R_{S_{0}/S_{1}}$ = 6, $L_{data}$ = 1000 bytes; (**f**) $R_{S_{0}/S_{1}}$ = 6, $L_{data}$ = 3000 bytes.

Figure 7 shows the saturation throughput on the basis of the number of nodes using the five different protocols. As the figure shows that, in the IEEE 1609.4, C-MAC and Q-VCI protocols, the throughput is getting lower when the number of nodes becomes larger. This is because the collision probability increases with the increase of number of nodes. For C-MAC and Q-VCI protocols, more time is set aside to CCH interval and the less time is given for SCH interval, while, for the IEEE 1609.4 protocol, the increasing number of nodes brings about more fierce competition on SCHs. In VeMAC protocol, due to the fixed frame length, the more nodes lead to the more number of successful SCH reservations, as we assumed in the front of this section, and thus the higher throughput on the SCHs. Since the proposed EQM-MAC protocol spends less time in *Safety interval* than IEEE 1609.4, VeMAC, C-MAC and Q-VCI protocols, its total throughput is higher than that of the other four protocols. Moreover, in the EQM-MAC protocol, an synchronization interval only contains both *Safety interval* and *WSA interval* for SCH reservations, and at the same time, the nodes can transmit non-safety messages on SCHs during the whole synchronization interval. Although the Q-VCI protocol uses higher data rate to transmit higher priority AC, the EQM-MAC protocol supports simultaneous transmissions on CCH and SCHs, and thus the EQM-MAC protocol always has the higher throughput than that of Q-VCI protocol under different $R_{S_{0}/S_{1}}$. On the other hand, the VeMAC protocol also can use SCHs during the whole synchronization interval, however, each node in VeMAC requires additional information to perform SCH reservation, thus the number of successful SCH reservations in VeMAC protocol is less than that in our proposed EQM-MAC protocol. Therefore, the EQM-MAC protocol can enhance the throughput and utilization of SCHs. For example, when $R_{S_{0}/S_{1}}$ = 2 and $L_{data}$ = 3000 bytes as shown in Figure 7d, EQM-MAC protocol can increase the average total throughput by 271%, 145%, 93% and 88%, compared with IEEE 1609.4, VeMAC, C-MAC and Q-VCI protocol, respectively. The proposed EQM-MAC protocol can provide efficient and QoS supported delivery of throughput, and the higher throughput requirement of higher priority service can thus be ensured.

**Figure 8:**
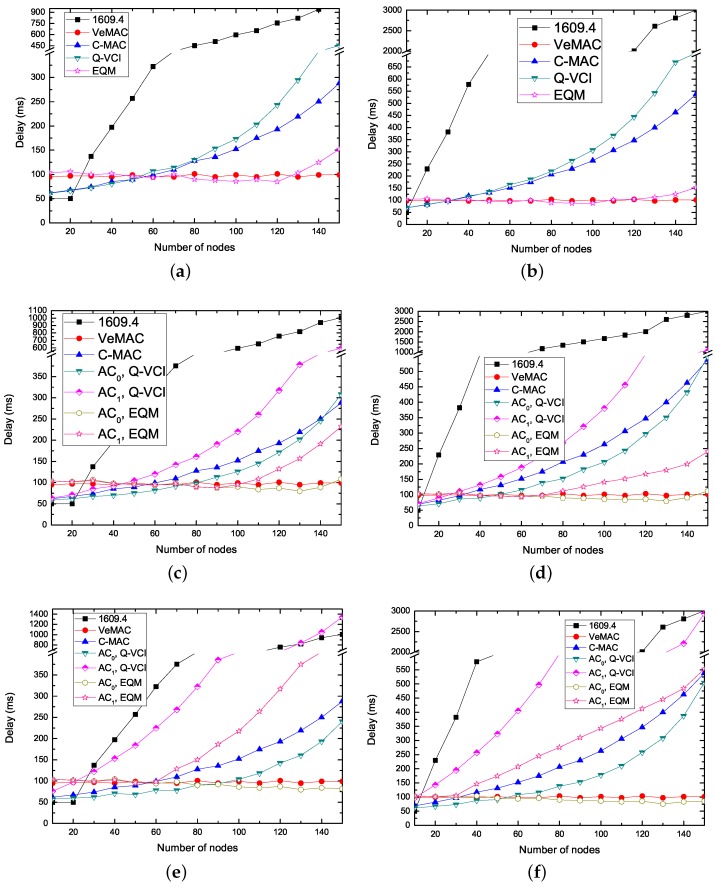
Non-safety packet delay versus the number of nodes for kinds of protocols ($N_{0}$ = $N_{1}$). (**a**) $R_{S_{0}/S_{1}}$ = 1, $L_{data}$ = 1000 bytes; (**b**) $R_{S_{0}/S_{1}}$ = 1, $L_{data}$ = 3000 bytes; (**c**) $R_{S_{0}/S_{1}}$ = 2, $L_{data}$ = 1000 bytes; (**d**) $R_{S_{0}/S_{1}}$ = 2, $L_{data}$ = 3000 bytes; (**e**) $R_{S_{0}/S_{1}}$ = 6, $L_{data}$ = 1000 bytes; (**f**) $R_{S_{0}/S_{1}}$ = 6, $L_{data}$ = 3000 bytes.

Figure 8 shows the average delay of non-safety packets versus the number of nodes using various protocols. The delay increases as the number of nodes increases except VeMAC protocol. This is because, for EQM-MAC, C-MAC and Q-VCI protocol, the collision probability increases when the number of nodes increases on the CCH, and thus the number of successful reservations decreases. Due to using contention-based mechanism on both CCH and SCH in IEEE 1609.4 protocol, the collision probability of transmission non-safety packets on SCHs increases with the number of nodes increasing. Since each node in VeMAC protocol can successful transmit a non-safety message in a frame, thus the delay of non-safety messages is constant and is about the half of a frame length. In most cases, the delay performance of packets of EQM-MAC protocol outperforms the others. The reason is that the EQM-MAC protocol can allow more time $T_{WI}$ for reservations than the VeMAC, C-MAC and Q-VCI protocol. Compared to the IEEE 1609.4 protocol, the EQM-MAC protocol employs coordination and contention-free transmissions on SCHs. We take $R_{S_{0}/S_{1}}$ = 2 and $L_{data}$ = 1000 bytes in Figure 8c for example to perform the following analysis. When the number of nodes is less than 40, the delay of nodes delivering packets with $AC_{0}$ ($AC_{1}$) of EQM-MAC protocol is slightly higher than that of the VeMAC, C-MAC and Q-VCI protocol, since the EQM-MAC protocol has longer $T_{WI}$ to wait before transmission than the other three protocols. However, as the number of nodes gets larger, EQM-MAC protocol displays significantly better performance for both $AC_{0}$ and $AC_{1}$ than that of the IEEE 1609.4, C-MAC and Q-VCI protocol. This is because, with the increase of the number of nodes, when using the IEEE 1609.4 protocol, the transmissions on SCHs are contention-based, and the contention becomes more intense, while, when using C-MAC and Q-VCI protocol, more time is allocated for safety messages and thus less time is left for non-safety messages. It can be also noted that, when the number of nodes is over 100, the delay of packets with $AC_{1}$ in the EQM-MAC protocol is higher than that in the VeMAC protocol. The reason is that, in the EQM-MAC protocol, the minimum CW for $AC_{0}$ is 32 and that for $AC_{1}$ is even larger, while in VeMAC protocol, the minimum CW for both ACs is always 32. The EQM-MAC protocol can ensure non-safety packets with lower transmission delay, and near-real time needs of some interactive entertainment applications can thus be guaranteed.

## 6. Conclusions

In this paper, we proposed an Efficient and QoS supported Multichannel MAC (EQM-MAC) protocol for VANETs. With the coordination of an RSU, the EQM-MAC protocol uses less time to deliver and reserve time slots for safety messages on the CCH, and thus, the time allocated for SCH reservation can be increased. The EQM-MAC protocol supports simultaneous transmissions on different SCHs during the whole SI, thus it enhances the saturation throughput and utilization of SCHs, and decreases the delay. On the other hand, the EQM-MAC protocol can provide QoS supported delivery on SCHs in terms of differentiated throughput and delay, by adaptively tuning the minimum CW for different non-safety service classes.

In our future work, based on the current work, we will design and develop an efficient inter-RSU communication mechanism that is able to reduce the effect of interference between vehicles in the overlapping regions. We will examine the effect of packet error caused by the hidden terminal and wireless channel impairments such as noise, fading and Non Line Of Sight (NLOS). We will also work on the analysis and design of the multichannel protocol for both safety-related and non-safety services under complex VANETs scenarios such as urban scenario. Finally, the multichannel performance in VANETs under multi-hop wireless conditions will be considered.

References yes

## Figures and Tables

**Table 1:** Summary of important notations.

Notation	Definition
SI	Synchronization Interval
CFI	Contention-Free Interval
VII	Vehicle Identification Interval
CLI	Coordination and Length Information
CP	Coordination Packet
RRTS	Reservation Request To Send
RFS	Request For Service
ACK/NACK	Acknowledgement / Non-acknowledgement
SUL	SCH Usage List
AC	Access Category
*N*	The total number of nodes
$N_{i}$	The number of nodes delivering non-safety packets with $AC_{i}$
β	The vehicle density on the highway (vehicles/m)
*R*	The RSU coverage
P(j,l)	The probability that *j* vehicles exist within length *l* of the highway
$n_{new}$	The number of newly entering vehicles during an SI
*M*	The number of lanes in each direction
$P_{i}$	The collision probability that a node delivering a WSA/RFS packet with $AC_{i}$ collides with other nodes when access the channel
$\tau_{i}$	The stationary probability that a node sends WSA/RFS packets belonging to $AC_{i}$ in a random time slot
$P_{i,suc}$	The probability that a node transmits WSA/RFS packets belonging to $AC_{i}$ to make a successful reservation
$P_{suc}$	The probability that a successful transmission of WSA/RFS packets occurs in a time slot
$P_{idle}$	The probability that the channel is idle
$P_{col}$	The probability that the channel collision occurs
*Z*	The time interval between two consecutive free time slots before a reservation is successfully made
$T_{suc}$	The duration of a successful reservation
$T_{col}$	The duration for a transmission collision when the node is performing a reservation
$T_{wsa}/T_{rfs}$	The duration for transmitting a WSA/RFS packet
$T_{ack}$	The duration for transmitting an ACK packet
δ	The duration of the propagation delay
$T_{reser}$	The duration from the time instant when a WSA/RFS becomes the head of the MAC queue to the time instant when a reservation is successful made or dropped due to reaching the maximum retransmission number
$T_{SI}$	The duration of an SI
$T_{CFI}$	The duration of CFI
$T_{VII}$	The duration of VII
$T_{WI}$	The duration of *WSA interval*
$T_{rrts}$	The duration of transmitting a RRTS packet
$T_{cp}$	The duration of transmitting a CP
$T_{data}$	The duration of a non-safety packet transmission on the SCH
$T_{h}$	The cost of MAC-layer header and physical-layer header
$T_{da}$	The duration of transmitting the payload of a non-safety packet
AIFS	The arbitration inter frame space
AIFSN	The AIFS number
SIFS	The duration of an Short Inter Frame Space (SIFS)
DIFS	The duration of a DIFS
σ	The duration of an idle time slot
$m^{\prime }$	The maximum times that the CW can be doubled
*m*	The maximum number of retransmissions
$W_{i,0}$	The minimum CW size of $AC_{i}$
$W_{i,j}$	The CW size of $AC_{i}$ in the *j*th stage
$b_{i,j,k}$	The stationary distribution of *k* state in *j*th stage for $AC_{i}$
$N_{sch}$	The number of available SCHs in VANETs
$G_{cch}$	The total number of successful SCH reservations on CCH
$G_{sch}$	The number of non-safety packets transmitted over all $N_{sch}$ SCHs during the whole SI
$L_{wsa}/L_{rfs}$	The payload of a WSA/RFS packet
$L_{ack}$	The payload of an ACK packet
$L_{data}$	The payload of a non-safety packet
$S_{sch}$	The total throughput obtained on $N_{sch}$ SCHs
$S_{i}$	The throughput obtained on $N_{sch}$ SCHs of nodes delivering non-safety packets with $AC_{i}$ (i=0,1,2,3)
$R_{S_{0}/S_{1}}$	The predefined throughput ratio of non-safety packets with $AC_{0}$ to $AC_{1}$
$\zeta_{i}$	The average number of successful transmissions belonging to $AC_{i}$ (i=0,1,2,3) on the SCHs of each node
$n_{i,suc}$	The number of successful transmissions belonging to $AC_{i}$ (${}_{i}=0,1,2,3$) on the SCHs
$T_{i,delay}$	The total transmission delay of non-safety packets belonging to $AC_{i}$ (${}_{i}=0,1,2,3$)
$R_{d}$	The transmission data rate on both CCH and SCHs
$N_{max}$	The maximum number of nodes exists in an RSU converge
$N_{masch}$	The maximum number of SCHs in vehicular environments
$N_{AC}$	The number of different ACs in vehicular networks
$CW_{max}$	The maximum CW size in vehicular networks
$m^{\prime \prime }$	The number of rounds that a node has to be experienced before it is identified by RSU

**Table 2:** Node’s SUL.

SCH	Available Slots
1	2, 7, 8
2	2, 4, 6
3	1, 3
4	1, 5

**Table 3:** SYSTEM PARAMETER FOR SIMULATIONS

Parameters	Values
Number of SCHs ($N_{sch}$)	4
Number of CCH	1
Date rate for each channel ($R_{d}$)	6 Mbps
RSU coverage (*R*)	300 m, 500 m
Number of lanes (*M*)	2-lane in each direction
Channel bandwidth	10 MHz
Channel model	Rayleigh fading
Pathloss exponent	4
Noise power	−100 dBm
Transmission power	23 dBm
Average of vehicle density (β)	0.02 to 0.3 vehicles/m
Vehicle velocity ($\left[V_{min},V_{max}\right]$)	[60, 100], [80, 120] km/h
$W_{0,0}$	32
$W_{1,0}$	32∼1024
$m^{\prime }$	5
m	10
MAC header	256 bits
PHY header	192 bits
The payload of a WSA/RFS packet ($L_{wsa}/L_{rfs}$)	216 bits + PHY header
The payload of an ACK packet ($L_{ack}$)	128 bits + PHY header
AIFSN for $AC_{0}$ (AIFSN[0])	6
AIFSN for $AC_{1}$ (AIFSN[1])	9
The duration of an SIFS (SIFS)	32 μs
The duration of an DIFS (DIFS)	58 μs
a slot time (σ)	13 μs
The propagation delay (δ)	1 μs
The duration of transmission an RRTS packet ($T_{rrts}$)	60 μs
The duration of transmission a Coordination Packet ($T_{cp}$)	100 μs
The duration of transmission a safety-related packet $T_{SaSlot}$	0.4 ms
Sending frequency of safety messages	10 Hz
The payload of a non-safety packet ($L_{data}$)	1000, 3000 bytes
Safety packet size	200 bytes
